# Diagnostic Value of Serum and Pleural Effusion Cancer Antigen 125 in Tuberculosis Diagnosis of Non-Cancer Patients: An Evidence-Based Case Report

**DOI:** 10.7759/cureus.42480

**Published:** 2023-07-26

**Authors:** Muhammad Maulana Wildani, Gurmeet Singh

**Affiliations:** 1 Department of Internal Medicine, Cipto Mangunkusumo Hospital - Faculty of Medicine Universitas Indonesia, Jakarta, IDN; 2 Division of Respirology and Critical Illness, Department of Internal Medicine, Cipto Mangunkusumo Hospital - Faculty of Medicine Universitas Indonesia, Jakarta, IDN

**Keywords:** blood, lung, ca-125 level, pleural effusion, tuberculosis

## Abstract

Tuberculosis can alter the permeability of the pleura and result in tuberculous pleural effusion. Clinical manifestation is similar to malignant pleural effusion making it challenging to distinguish. Tumor marker cancer antigen 125 (CA-125) level of pleural fluid could be an alternative in diagnosing tuberculous pleural effusion. We reported a case of a 41-year-old female with shortness of breath and a history of chronic kidney disease, acute decompensated heart failure, and community-acquired pneumonia. The patient underwent tuberculosis examination and yielded negative result, yet the serum CA-125 examination yielded positive result. A literature search was performed on electronic databases with appropriate search terms based on the established clinical question and a total of three cross-sectional studies were selected based on the eligibility criteria. CA-125 level of pleural fluid sample was found to have a good diagnostic value compared to the blood serum sample. However, further research is necessary to determine a proper cut-off value for a significant result.

## Introduction

Around 10 million people are diagnosed with tuberculosis (TB) worldwide, putting it as the second leading infectious killer worldwide. Despite the effort made to control TB, in 2020, an estimated 10 million people were diagnosed with TB worldwide. There are numerous examination modalities that had been developed to diagnose TB [1,2]. However, clinical manifestation is similar to malignant pleural effusion making it challenging to distinguish. Cancer antigen 125 (CA-125) is a tumor marker that is expressed by the epithelial ovarian, and is also found in some normal and inflammatory cells [3]. An elevated CA-125 presents in a variety of malignant and benign conditions, such as endometriosis, ovarian cysts, hepatic cirrhosis, pleural effusions, and extrapulmonary or pulmonary tuberculosis (PTB) [3,4]. The rationale of elevated CA-125 in TB is because CA-125 could be found in normal mesothelial lung cells and normal bronchial epithelial cells. A destruction that occurred to these cells causes CA-125 to be released, and found to be increased in the serum. Studies have found the relation between elevated serum CA-125 and PTB, thus measuring CA-125 level may be an adjuvant tool in diagnosing TB and is useful in the follow-up patients after receiving antituberculous treatment [4].

An inflammatory or non-inflammatory mechanism may result in pleural effusion. Increased hydrostatic pressure, decreased oncotic pressure, and changes in lymphatic drainage play an important role in the mechanism of pleural effusion. In non-inflammatory situations, the pleural space is mostly made up of lymphocytes, macrophages, and endothelial cells, with tiny amounts of protein, lactate dehydrogenase (LDH), and transudate fluid. However, changes in the permeability of the pleura occur as a result of infection. Recognizing the underlying cause of pleural effusion can be difficult due to its similarity to the clinical manifestation of malignancy cases. The clinical manifestation of the accumulation process of pleural fluid in tuberculosis infection is found to be just mild and chronic [5]. Nonetheless, detecting the cause of pleural effusion is crucial for choosing the appropriate treatment. A popular procedure in this regard is the cytological evaluation of pleural effusion. Its sensitivity ranges from 43% to 83%, which is far from ideal. Thoracoscopy is then used to make the diagnosis in pleural effusion instances with negative cytological results. This approach may be too invasive for patients in poor physical conditions [6]. A useful and minimally invasive method for identifying the cause of pleural effusion is needed to address these issues, such as measuring the concentration of tumor markers like CA-125 through thoracocentesis. We reported a case of a 41-year-old woman who presented with shortness of breath and a history of chronic kidney disease, acute decompensated heart failure, and community-acquired pneumonia and yielded positive results in its serum CA-125 measurement.

## Case presentation

**Figure 1:**
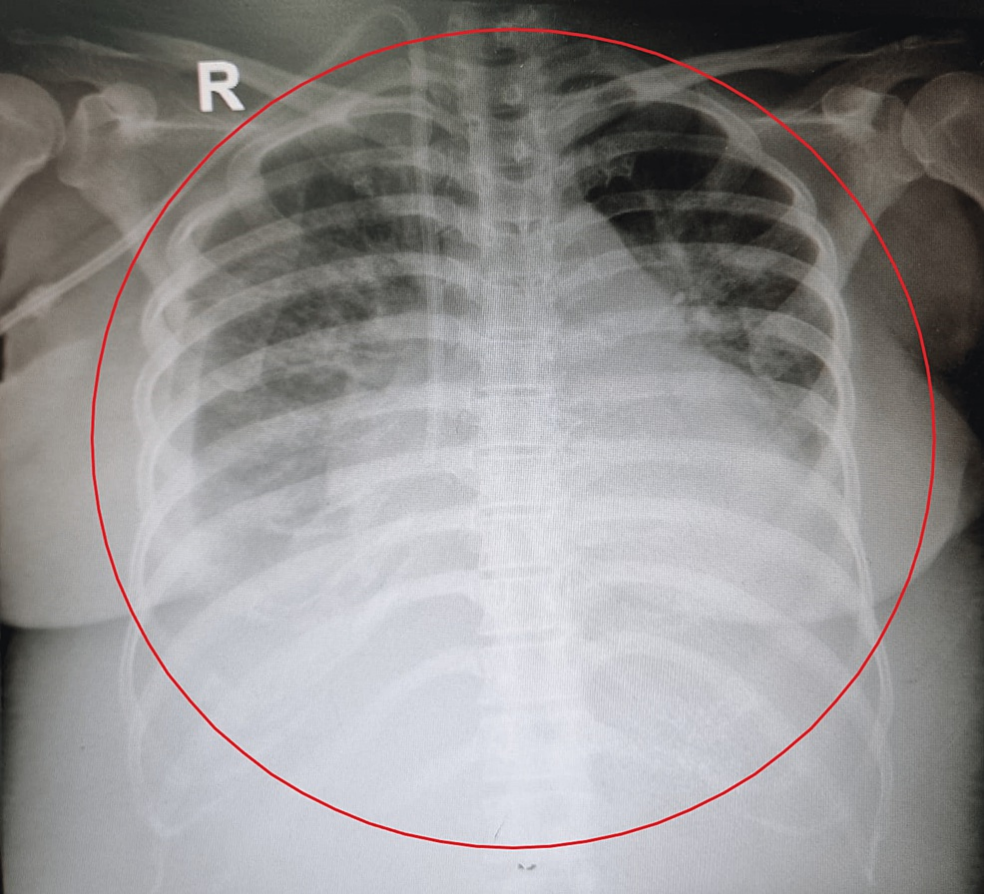
Plain radiography of the patient

A 41-year-old woman was admitted to the hospital due to shortness of breath that worsened one day before hospital admission. The patient had felt shortness of breath in the past seven months prior to hospital admission, in conjunction with dyspnea *d’effort*, paroxysmal nocturnal dyspnea, orthopnea, and bilateral leg edema. General examination revealed the patient to be short of breath (respiratory rate 18 times per minute), had high blood pressure (168/74 mmHg), anemic conjunctiva, and bilateral pitting edema. A double-lumen catheter was attached to the patient's right jugular vein as the patient was planned to undergo hemodialysis. Systemic examination revealed bilateral coarse crackles, percussion was not done because the patient coughs every time a percussion is done. Abdominal examination revealed ascites. Laboratory evaluation (Tables 1, 2) revealed an impression of normochromic normocytic anemia, leukocytosis, neutrophilia, high urea and creatinine levels, chronic kidney disease grade 5, and glucose level fluctuation above the normal range. On radiological examination, the impression of cardiomegaly, bilateral pleural effusions, infiltrates in both lung fields, and a central venous catheter that ends on the superior vena cava was obtained (Figure 1). The patient denies a history of having tuberculosis infection, supported by an undetected Mycobacterium tuberculosis (MTB) using GeneXpert MTB/RIF. The serum CA-125 test was later requested to rule out lung tuberculosis in this patient. The result of the serum CA-125 test was found to be increased than the normal range (Table 1). The pleural effusion CA-125 test was planned to confirm the findings, however, the patient refused to the procedure. The patient was later diagnosed with pleural tuberculosis and treated with antituberculosis. In addition, the patient was also diagnosed with hypertension, diabetes mellitus type 2, and heart failure. The pleural effusion improved after three days of antituberculosis treatment and dyspnea was no longer present. The patient was discharged afterwards.

**Table 1:** Laboratory examination results CA-125: cancer antigen-125; eGFR: estimated glomerular filtration rate; NLCR: neutrophil-lymphocyte count ratio.

Examination	Result	Unit	Reference Value
COMPLETE BLOOD COUNT			
Hemoglobin	10.3	g/dL	12.0 – 15.0
Hematocrit	30.9	%	36.0 – 46.0
Erythrocyte	3.58	10^6^/μL	3.80 – 4.80
Mean Corpuscular Volume	86.3	fL	83.0 – 101.0
Mean Corpuscular Hemoglobin	28.8	Pg	27.0 – 32.0
Mean Corpuscular Hemoglobin Concentration	33.3	g/dL	31.5 – 34.5
Thrombocyte	245	10^3^/μL	150 – 410
Leukocyte	12.35	10^3^/μL	4.00 – 10.00
DIFFERENTIAL COUNT			
Basophil	0.4	%	0 – 2
Eosinophil	16.0	%	1 – 6
Neutrophil	64.1	%	40.0 – 80.0
Lymphocyte	11.7	%	20 – 40
Monocyte	7.8	%	2 – 10
Red Cell Distribution Width – Coefficient of Variation	15.4		11.6 – 14.0
Red Cell Distribution Width – Standard Deviation	48.3		
NEUTROPHIL-LYMPHOCYTE COUNT RATIO			
Neutrophil Count	7.92	10^3^/μL	1.70 – 7.50
Lymphocyte Count	1.45	10^3^/μL	1.00 – 3.20
NLCR	5.46		
RENAL FUNCTION			
Creatinine (Blood)	4.50	mg/dL	0.55 – 1.02
eGFR	11.4	mL/min/1.73m^2^	94.00 – 142.00
Blood Urea Nitrogen	87.7	mg/dL	15 – 40
ELECTROLYTE			
Sodium (Blood)	139	mEq/L	136 – 145
Potassium (Blood)	4.0	mEq/L	3.5 – 5.1
Chloride (Blood)	106.3	mEq/L	98.0 – 107.0
CA-125			
Serum CA-125	125	U/mL	0 – 35

**Table 2:** Blood glucose level examination result

BLOOD GLUCOSE LEVEL			
Date of Examination	Result	Unit	Reference Value
3^rd^ June 2022	147	mg/dL	60 – 180
10^th^ June 2022	284	mg/dL	60 – 180
11^th^ June 2022	187	mg/dL	60 – 180
12^th^ June 2022	68	mg/dL	60 – 180
13^th^ June 2022	180	mg/dL	60 – 180

## Discussion

**Figure 2:**
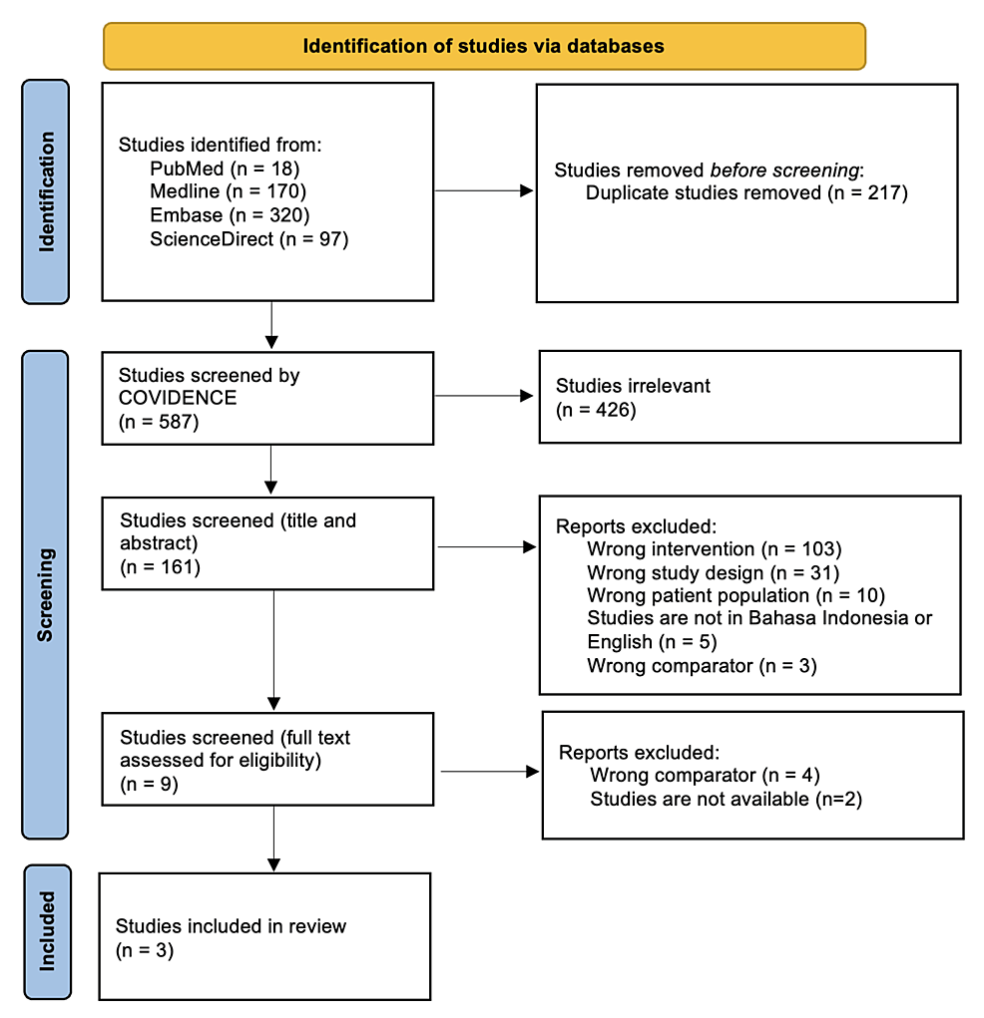
Flowchart of the article selection process

A comprehensive literature search was performed with multiple electronic databases, such as PubMed, Medline, Embase, and ScienceDirect in July 2022. Search terms included variations of “tuberculosis”, “CA-125”, and “pleural effusion”. The inclusion criteria were a systematic review of cross-sectional or cross-sectional studies, patients with clinical manifestation of TB, and studies that compared CA-125 levels of pleural fluid samples and blood samples. The exclusion criteria were patients with malignancies and studies with CEBM Level of Evidence 4 or lower. The initial search yielded 804 articles, and later three cross-sectional studies were included in the narrative review after the exclusion process (Figure 2).

Characteristics of included studies are shown in Table 3.

**Table 3:** Summary of included studies CA-125: cancer antigen-125; Sn: Sensitivity; Sp: Specificity; PPV: Positive predictive value; NPV: Negative predictive value; AUC: Area under the curve.

Author	Population	Intervention	Comparison	Outcome
Gu et al. (2016) [7]	35 patients with tuberculous pleural effusion (TPE)	Level of CA-125 of pleural fluid	Level of CA-125 of blood serum	Diagnostic values (Sn, Sp, PPV, NPV, AUC, accuracy)
Lin and Ni (2022) [8]	15 patients with tuberculous pleural effusion (TPE)	Level of CA-125 of pleural fluid	Clinical manifestation Chest radiograph showing pleural-based opacity obscuring the diaphragm	Diagnostic values (Sn, Sp, PPV, NPV, AUC, accuracy)
Hoshy et al. (2016) [9]	20 patients with tuberculous effusion	Level of CA-125 of pleural fluid	Ziehl Neelsen smear and/or culture of pleural fluid/ or pleural biopsy showing confirmed TB diagnosis	Diagnostic values (Sn, Sp, PPV, NPV, AUC, accuracy)

There is scarce evidence in comparing the pleural fluid CA-125 level with blood serum in diagnosing TB. Currently, there are no systematic reviews and meta-analyses included in this report.

In the study of Gu et al., a cross-sectional study was conducted on 35 patients with a confirmed diagnosis of tuberculous pleural effusions. This study was the only study that compared diagnostic values of CA-125 level which samples from pleural fluid and blood serum [7]. The study shows CA-125 levels of pleural fluid sample have a sensitivity of 67.37%, specificity of 74.29%, accuracy of 66.92%, and AUC of 0.75. As for the CA-125 levels of blood serum sample, it has a sensitivity of 33.68%, specificity of 88.57%, accuracy of 48.46%, and AUC of 0.58. The cut-off values for the pleural fluid sample were found to be 644.30 U/ml with a positive predictive value of 90.62% and a negative predictive value of 43.94%. As for the blood serum sample, the cut-off values were found to be 151.55 U/ml with a positive predictive value of 88.89% and the negative predictive value of blood serum samples is 32.98%. Compared to both, the positive and negative predictive value of blood serum samples is lower than pleural fluid samples. To conclude, this study shows pleural effusion CA-125 levels are more suitable to rule in the diagnosis of TB as the cause of pleural effusion, rather than to rule it out. Another result from the study is, generally, CA-125 level for TB diagnosis is more accurate using pleural fluid samples than blood serum samples [7].

The study of Lin and Ni, distinctly from the first study, compares CA-125 levels from pleural fluid samples and thoracocentesis examination analysis on 15 patients diagnosed with tuberculous pleural effusion. The diagnosis to distinguish from the diagnostic test was made through thoracocentesis and the pleural fluid was then proceeded to biochemical and/or microbiological examination analysis. The cut-off value of this study was set at 1594.35 U/ml. The diagnostic values were found to be a sensitivity of 13.3%, specificity of 89.5%, positive predictive value of 46.3%, negative predictive value of 60.2%, accuracy of 59%, and AUC of 0.344. In conclusion, the CA-125 level of the pleural fluid sample alone from this study shows a little significance in the diagnosis of pleural effusion caused by TB infection, but is more able to identify those who do not have the disease [8].

In the study of Hoshy et al., a total of 20 patients with a confirmed diagnosis of tuberculous effusion were included. The cut-off value of this study was 900 U/mL. The diagnostic values were sensitivity 74.1%, specificity 76.9%, positive predictive value 70%, negative predictive value 33.3%, and AUC 0.486. Hence, the diagnostic value of CA-125 level from pleural fluid samples has a slight similarity for its sensitivity and specificity, but keep in mind for probable false negative results [9].

The results of the three studies reviewed showed a large difference between the results of Gu et al. study and the other two studies. The positive predictive value and negative predictive value in Lin and Ni study and Hoshy et al. study showed a significant difference and are contrary to each other. The AUC in the study of Lin and Ni and Hoshy et al. showed a score of <0.5, which means the test quality was found to be unsatisfactory [8,9]. However, the study of Gu et al. had an AUC score of >0.7, meaning the test quality is good [7]. On the other hand, the three studies used different cut-off values for CA-125 level, where an unrepresentative cut-off value can also affect the sensitivity, specificity, and accuracy of the test [7-9,10]. In addition, another diagnostic study in ovarian carcinoma patients reported that there are some possible confounding factors that could affect the accuracy of the test, such as another malignancy, hypertension, and diabetes mellitus. Both hypertension and diabetes mellitus were found to play a role in influencing CA-125 performance by having a significant decrease in CA-125 concentration, causing a big number of false negative results and a significant decrease in sensitivity [10].

Despite no evaluation for CA-125 level of pleural fluid in this case report, other studies have reported that CA-125 level in tuberculous pleural effusion was significantly higher compared to other infections, yet lower than malignancy cases [11]. Estimating TB infection may be very difficult to distinguish from other etiology of the pleural effusion if only observed by unidentified and unclassical history. Thus, other diagnostic tests that may also be required could be avoided by measuring the CA-125 level. Lastly, this study concluded that the accuracy and other diagnostic values of CA-125 level were found to be preferable using pleural fluid samples than blood serum samples [7-9].

## Conclusions

CA-125 level of pleural fluid sample can be potentially useful for diagnosing tuberculous pleural effusion due to its high accuracy, compared to using a blood serum sample. Further research between measurements of CA-125 level using pleural fluid samples needs to be done to further evaluate its potential use. A cut-off value for the increase of CA-125 level that positively shows tuberculosis infection as the etiology of pleural effusion also needs to be investigated further to yield a better sensitivity and specificity of the test. Predisposing medical conditions such as hypertension and diabetes mellitus may act as confounding factors and should be taken into account when interpreting the result of CA-125 examination. Despite the examination of serum CA-125 level yielding promising result in diagnosing tuberculosis of this patient, this case report was not able to provide the data of pleural effusion CA-125 level as the patient did not provide consent to the procedure.
